# Tomato Juice Consumption Modifies the Urinary Peptide Profile in Sprague-Dawley Rats with Induced Hepatic Steatosis

**DOI:** 10.3390/ijms17111789

**Published:** 2016-10-26

**Authors:** Gala Martín-Pozuelo, Rocío González-Barrio, Gonzalo G. Barberá, Amaya Albalat, Javier García-Alonso, William Mullen, Harald Mischak, María Jesús Periago

**Affiliations:** 1 Department of Food Technology, Food Science and Nutrition, Faculty of Veterinary, Regional Campus of International Excellence “Campus Mare Nostrum, University of Murcia, Murcia 30071, Spain; rgbarrio@um.es (R.G.-B.); fjgarcia@um.es (J.G.-A.); mjperi@um.es (M.J.P.); 2 Biomedical Research Institute of Murcia (IMIB-Arrixaca-UMU), University Clinical Hospital “Virgen de la Arrixaca”, University of Murcia, Murcia 30120, Spain; 3 Department of Soil and Water Conservation, CSIC-CEBAS, Campus Universitario, Murcia 30100, Spain; gbarbera@cebas.csic.es; 4 Institute of Cardiovascular and Medical Science, University of Glasgow, Glasgow G12 8TA, UK; amaya.albalat@stir.ac.uk (A.A.); william.mullen@glasgow.ac.uk (W.M.); mischak@mosaiques-diagnostics.com (H.M.); 5 Mosaiques Diagnostics GmbH, Hannover 30659, Germany

**Keywords:** non-alcoholic fatty liver disease (NAFLD), tomato, biomarkers, peptidome, proteome, urine, capillary electrophoresis coupled to a mass spectrometer (CE-MS), diagnosis, liver

## Abstract

Non-alcoholic fatty liver disease (NAFLD) is the most common liver disorder in Western countries, with a high prevalence, and has been shown to increase the risk of type 2 diabetes, cardiovascular disease (CVD), etc. Tomato products contain several natural antioxidants, including lycopene—which has displayed a preventive effect on the development of steatosis and CVD. Accordingly, the aim of the present work was to evaluate the effect of tomato juice consumption on the urinary peptide profile in rats with NAFLD induced by an atherogenic diet and to identify potential peptide biomarkers for diagnosis. Urine samples, collected weekly for four weeks, were analyzed by capillary electrophoresis (CE) coupled to a mass spectrometer (MS). A partial least squares-discriminant analysis (PLS-DA) was carried out to explore the association between differential peptides and treatments. Among the 888 peptides initially identified, a total of 55 were obtained as potential biomarkers. Rats with steatosis after tomato juice intake showed a profile intermediate between that of healthy rats and that of rats with induced hepatic steatosis. Accordingly, tomato products could be considered as a dietary strategy for the impairment of NAFLD, although further research should be carried out to develop a specific biomarkers panel for NAFLD.

## 1. Introduction

Non-alcoholic fatty liver disease (NAFLD) is the most common liver disorder in Western countries and has a high prevalence, affecting between 14% and 24% of the population and reaching higher incidence (25%–75%) in cases of obesity and type 2 diabetes individuals. Traditionally, NAFLD has affected the adult population; however, it is extending to children and adolescents, due to the increased prevalence of obesity in these sub-populations [1,2]. Obesity, type 2 diabetes, dyslipidemia and hypertension are the most important risk factors, and NAFLD is considered the hepatic manifestation of metabolic syndrome. The hallmark of NAFLD is hepatic lipid accumulation, mainly triglycerides, in the absence of significant ethanol consumption or viral hepatitis. It covers a spectrum of pathologies, ranging from hepatic steatosis to steatohepatitis (NASH), fibrosis and even cirrhosis. Although significant progress in understanding the pathogenesis of NAFLD has been achieved in recent years, the mechanisms leading to liver steatosis and further progress to NASH still remain unclear [3,4].

Two steps or hits have been proposed for the pathophysiology of NAFLD and NASH: the first hit is due to the triglycerides accumulation as a consequence of insulin resistance; and the second hit includes oxidative stress, lipid peroxidation, increased cytokine production and inflammation, resulting in NASH [5]. Although the “two hit hypothesis” is the most supported theory, currently under consideration is a “multiple parallel hits hypothesis”, which suggests that overlapping among insulin resistance, hepatic de novo lipogenesis and subsequent hepatocyte injury, as well as the effects of some candidate genes, could contribute to the progression from simple steatosis to NASH [6,7].

In addition, patients with NAFLD show an important risk of development of cardiovascular disease (CVD), mainly associated with the abnormalities in lipid and lipoprotein metabolism accompanied by chronic inflammation, and even by oxidative stress. In fact, the current evidence raises the possibility that NAFLD may not only be a marker, but also an early mediator of atherosclerosis [8,9].

Currently, lifestyle modification, including changes in dietary habits, is the most accepted treatment for NAFLD [10,11,12]. In this regard, the Mediterranean diet has been suggested as the most appropriate dietary strategy for this pathology, mainly due to the high consumption of plant-based foods and low intake of saturated fats and refined sugars [13]. Tomato products are a dietary source of natural antioxidants such as vitamins C and E, polyphenols, β-carotene and, especially, lycopene, the most abundant carotenoid in this fruit [14,15]. Previous studies have shown that the consumption of tomatoes and tomato products strengthens the antioxidant system and inhibits lipid peroxidation in humans [16,17]. Scientific evidence suggests that the role of lycopene as an antioxidant agent in the prevention of CVD is related to the effect of this carotenoid on lipoprotein metabolism, decreasing total cholesterol and the content and oxidation of LDL-cholesterol [17,18,19]. Moreover, lycopene has been found to be a most effective antioxidant for liver health [20], showing different beneficial effects on liver metabolism in rats with induced NAFLD. Related to this, different authors have described, in animal models, a preventive effect of tomato consumption on steatosis, increasing mitochondrial and peroxisomal fatty acid oxidation [21,22,23,24], that could prevent the development of NASH due to the inhibitory effect of lipid peroxidation in the liver tissue [25,26].

In our research group, we have shown that the consumption of tomato juice by rats with NAFLD induced by a high-fat diet ameliorated the steatosis, improving the metabolic pattern in the animals—which reached a state more similar to that of healthy rats [21,24]. In these investigations, invasive methodologies—based on the analysis of plasmatic biomarkers and the amino acid profile and gene expression of the liver—were applied to evaluate the effect of the accumulation of lycopene on NAFLD. Recently, technologies based on proteome and peptidome analysis of biological fluids are becoming targets for disease diagnosis, since they provide large amounts of information on the physiological state of an organism. In this regard, urine is easy to collect in large quantities and it is more stable and less complex than blood, making it a suitable fluid for peptide biomarkers detection [27,28]. Capillary electrophoresis coupled to a mass spectrometer (CE-MS) has been shown to be an excellent platform to identify urinary biomarkers for diagnosis [29,30,31].

Currently, liver biopsy remains the gold standard for the diagnosis of NAFLD. However, liver biopsy is an invasive procedure not fit for general screening given its associated costs, risks and sampling errors, as the liver is not necessarily uniformly affected by this pathology [32]. Moreover, the high prevalence of NAFLD, especially in high-risk sub-populations, makes the employment of liver biopsy difficult due to its low-throughput nature; thus, there is a crucial need to discover new biomarkers for the diagnosis and evaluation of the stage of NAFLD through non-invasive methods. Using proteomics, several studies have revealed a number of serum proteins for the diagnosis of NAFLD [33,34]. However, the blood proteome is highly complex, with an extensive mass range, and the collection of blood is an invasive procedure [31].

Taking all factors into consideration, the aim of this investigation was to identify potential biomarkers for the diagnosis of NAFLD, using a peptidomic approach, and to evaluate if the intake of tomato juice modifies the urinary peptide profile in Sprague-Dawley rats with hepatic steatosis induced by a high-fat diet, in comparison with healthy animals. The urinary peptide profile was analyzed using CE-MS, in order to discover specific biomarkers for the diagnosis of this pathology.

## 2. Results

In terms of the biochemical parameters, total cholesterol was higher in hypercholesterolemic and high-fat diet (H) than in standard diet (N) groups (Table 1). As expected, total cholesterol, LDL-cholesterol and triglycerides were significantly higher in the rats with induced steatosis (hypercholesterolemic and high-fat diet and water (HA) and hypercholesterolemic and high-fat diet and tomato juice (HL) than in the rats of groups standard diet and water (NA) and standard diet and tomato juice (NL). All the changes in the lipid profile were related to diet H, but some changes were also associated with the intake of tomato juice in the NL group (Table 1). These changes show the dyslipidemia associated with NAFLD, which was also confirmed by the increase in the alanine transaminase (ALT) and aspartate transaminase (AST) enzymes, in rats fed the H diet (Table 1). In a previous study [24], we confirmed by histological examination the steatosis grade of 2 or 3 in these animals, according to the classification of Brunt et al. [35].

**Figure 1:**
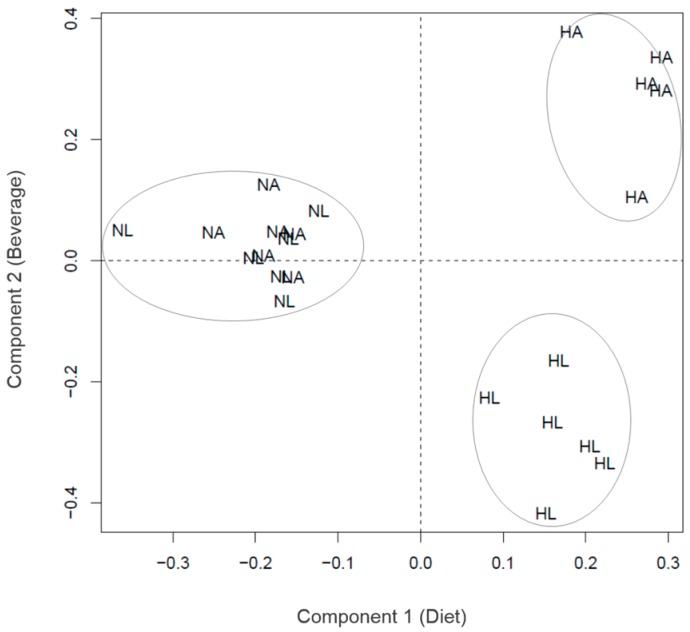
sPLS-DA model representing the four experimental groups in two components according to their peptide profile. NA: standard diet and water; NL: standard diet and tomato juice; HA: hypercholesterolemic and high-fat diet and water; HL: hypercholesterolemic and high-fat diet and tomato juice; Component 1: diet and Component 2: beverage.

**Figure 2:**
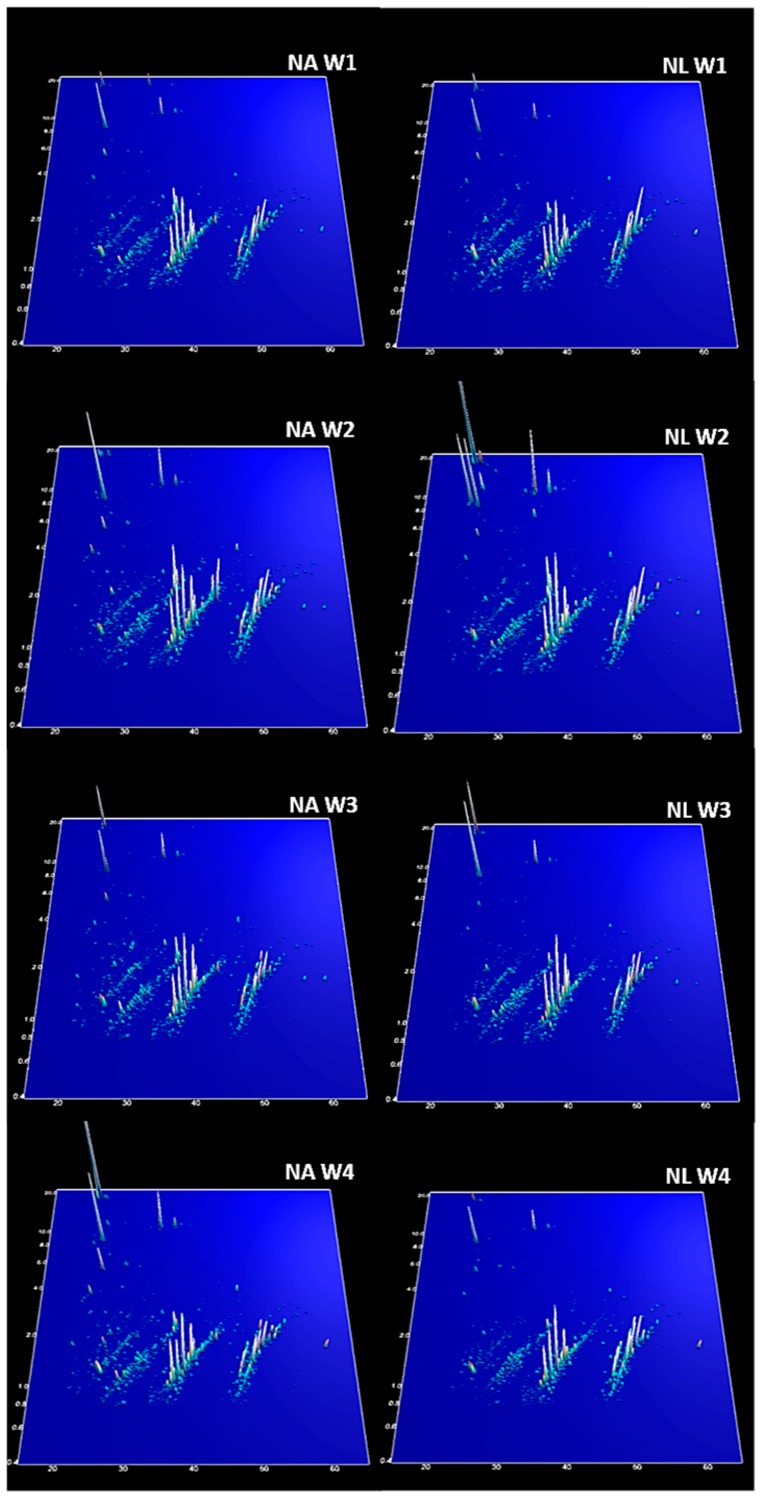
Urinary peptide fingerprints of groups control, NA and NL. The CE migration time (min) (***X*-axis**) is plotted against the molecular mass (kDa) on a logarithmic scale (***Y*-axis**). The ***Z*-axis** represents the mean signal intensity. W1–4: Week 1–4.

**Figure 3:**
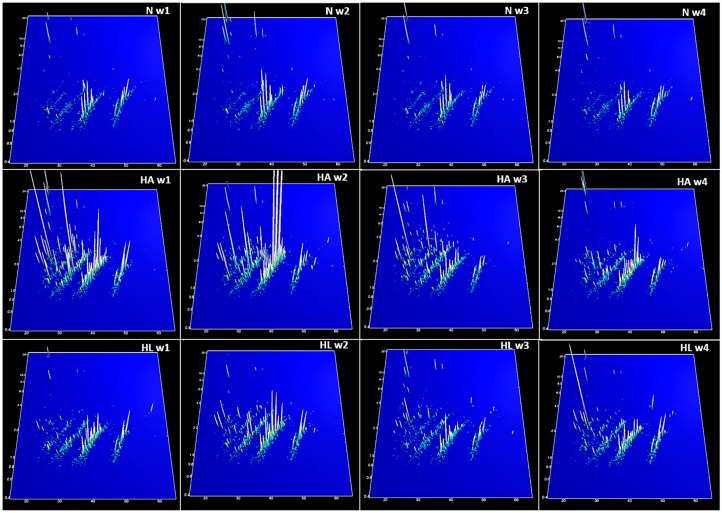
Compiled CE-MS urinary peptide fingerprints of groups N, HA and HL over the weeks. The CE migration time (min) (***X*-axis**) is plotted against the molecular mass (kDa) on a logarithmic scale (***Y*-axis**). The ***Z*-axis** represents the mean signal intensity. N: standard diet; CE: capillary electrophoresis.

**Figure 4:**
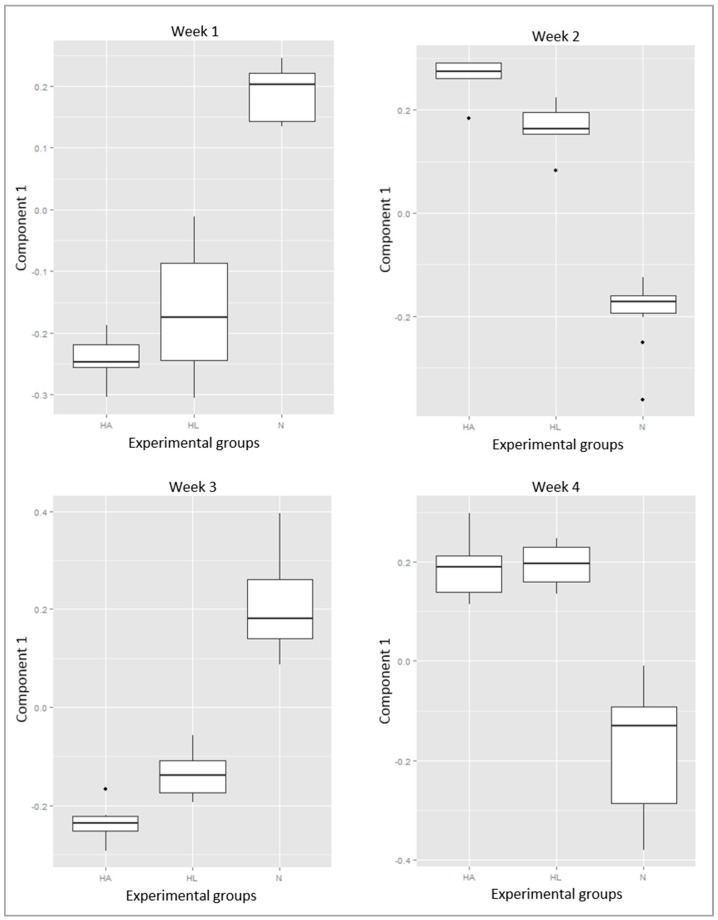
Box-and-whisker plots of component 1 for the three experimental groups (N, HA and HL) over four weeks.

As explained in the Methods, the best sparse partial least squares-discriminant analysis (sPLS-DA) classification (lowest error rate) was for the data organized in three groups (N, HA, HL). Figure 1 (the model for Week 2, with two components and 15 peptides) is an example of a typical result of the sPLS-DA, with the three groups clearly separated (NA + NL, HA, HL; Figure 1). The first component segregates N (NA + NL) rats from HL and HA, but it should be noted that the HL scores (coordinates on component 1) are intermediate between N (NA + NL) and HA. The second component segregates HA from HL. The pattern of this example is basically repeated in all the models, and shows that the peptide profile was mainly conditioned by diet, H (HA and HL) being separated from N (NA + NL), although the ingestion of tomato juice shifted the HL profile closer to that of N (NA + NL). The second component is related mostly to changes in the peptide profile associated with the ingestion of tomato juice, but within diet H. The fingerprinting images are consistent with the interpretation suggested by the sPLS-DA models. The peptide profile was similar for groups NA and NL (Figure 2). Interestingly, on the fingerprinting images the intermediate position of HL between the control group (N) and the high-fat group (HA) (Figure 3) is clear from Weeks 1 to 3 but vanishes at Week 4. This pattern is also consistent with the sPLS-DA results. Figure 4 shows boxplots of the scores on component 1, which separates the animals according to the diet: the intermediate position of HL is maintained in Weeks 1 to 3 but vanishes at Week 4.

The urinary polypeptide fingerprints of the group with hepatic steatosis but without tomato juice consumption (HA) were very different to those of the other groups, showing a high complexity during the first three weeks—especially due to the high relative abundance of most of the peptides and the presence of high-molecular-weight peptides, as shown in the proteomic fingerprints (Figure 3).

Initially, 888 peptides were obtained after processing the urine samples. However, according to the sPLS-DA models, only 55 peptides (changing a long time) can be considered potential biomarkers (Table 2) according to the protocol established in the Methods. These peptides were present at a frequency ≥30% in at least one group (control or cases), a criterion used in other biomarker studies [36].

The protein identity was derived by matching the amino acid sequences against a protein database [36]. Table 2 shows the week, the identified and named proteins, the mass and the migration time of a peptide in the CE-MS analysis, as well as the mean relative abundance, the frequency of occurrence of each peptide and the fold change of groups HA and HL versus group N.

Overall, most of the 55 peptides showed clear differences between the groups fed with the standard diet or high-fat diet, as shown by the fold change values (Table 2). However, some peptides showed greater increases or decreases in the hepatic steatosis group with respect to group N, group HL being closer to the healthy group over the weeks (Figure 4).

With regard to the behavior of specific proteins, apolipoprotein A-IV (peptide 13451) was found to decrease in group HA, compared to groups N and HL, in Weeks 1 and 3. Collagen α-1(I) chain (peptide 4970) and collagen α-1(II) chain (peptide 11153) showed a clear increase in group HA in Week 2, whilst their values in group HL were closer to those of group N. Fibrinogen α chain precursor (peptide 7243), extracellular superoxide dismutase (Cu-Zn) (peptide 9955) and proline-rich protein (peptide 14976) showed the same tendency in Week 2 as the above-mentioned collagen α chains. Uromodulin (peptide 14544) had greater abundance in the H groups, being especially increased in group HA, and was not detected in group N at Week 3. In contrast, in Week 3, transketolase (peptide 8116) and l-lactate dehydrogenase B chain (peptide 14576) showed important decreases in the H groups, being lower in rats that did not drink tomato juice. However, it is important to remark that in Week 4 only two peptides, 6975 (collagen α-1(I) chain) and 11644 (collagen α-1(I) chain precursor), were identified as classificatory peptides.

## 3. Discussion

The CE-MS approach is rapid, sensitive and automated [29]. In addition, this platform allows the detection of differences between the urinary proteomes from healthy and unhealthy individuals; therefore, it is a useful tool for the diagnosis and prevention of diseases. For this reason, one of the objectives of this work was to discover potential biomarkers associated with hepatic steatosis, since, to the best of our knowledge, there is currently no specific panel of urinary biomarkers for NAFLD. Taking into consideration that the prevalence of NAFLD is dramatically increasing and that its diagnosis is mainly based on a liver biopsy, proteomics appears an interesting approach not only for the diagnosis of NAFLD but also to allow better understanding of its pathogenesis, employing a non-invasive method [37].

The urinary peptide fingerprints of the three groups (N, HA and HL) (Figure 3), as well as the sPLS-DA models (Figure 4), suggest that rats fed the H diets showed changes related to their physiological condition, since rats of groups HA and HL had steatosis with associated clinical symptoms, like dyslipidemia and increased activity of the enzymes ALT and AST. However, it is noteworthy that the rats of group HL showed a peptide profile in a position intermediate between those of groups HA and N along the three first weeks. This suggests that tomato juice intake could have modified the urinary peptide profile of rats fed with the high-fat diet, leading to a status closer to that of the healthy group. This tendency is also supported by Figure 4, where group HL is found in a similar situation from Weeks 1 to 3. This may be due to the tomato juice consumption triggering a protective effect on the pathophysiology of hepatic steatosis during the three first weeks, this effect having been overcome by the mechanisms of the pathogenesis at the end of the study (Week 4).

The positive effect of tomato juice consumption, and the consequent accumulation of lycopene in the liver, has been described for these rats in previous studies conducted by our research group. Bernal et al. [21] reported a complex effect of lycopene from tomato juice, showing: (a) alleviation of amino acid depletion; (b) recovery of the redox balance in the liver; and (c) an increase in l-carnitine, which could indicate an improvement in the transport of fatty acids into the mitochondria. Martín-Pozuelo et al. [24] described a decrease in urinary isoprostranes and an over-expression of genes related to mitochondrial and peroxisomal fatty acid oxidation in rats with steatosis that had drunk tomato juice (HL group). In general, other authors, using different experimental designs, have reported a beneficial effect of tomato or lycopene consumption on NAFLD, by reduction of the oxidative stress and also improvement of the lipid metabolism, exerting a preventive effect on the progression to NASH [22,23,25].

In our present work, most of the 55 discriminant peptides were able to show a clear difference between animals fed with the standard diet and those receiving the high-fat diet (N and H groups), as can be observed in the fold change values included in Table 2. Some interesting changes were observed in several proteins, identified from peptides analysed in the urine samples by CE-MS, whose levels were increased or reduced in group HA compared to group N, group HL showing, in most of these cases, levels closer to those of the healthy group.

Apolipoprotein A-IV is secreted in the small intestine, to absorb dietary fats, and it is also involved in glucose homeostasis and the reverse transport of cholesterol and lipids through chylomicrons and high density lipoproteins (HDL). In addition, it possesses important antioxidant and anti-inflammatory properties. For these reasons, the circulatory levels of this protein have been considered a target in the diagnosis and treatment of CVD, as well as diabetes and obesity [38]. Moreover, different proteomic studies [39] have shown that a deficiency of plasmatic apolipoproteins is associated with a higher prevalence of NAFLD. This is in concordance with our findings, since the abundance of apolipoprotein A-IV was lower in urine samples from the H groups (fold change −2.20 in HA and −1.72 in HL, Table 2) compared to group N, reflecting the fact that this apolipoprotein is mainly present in HDL lipoproteins, whose plasma levels were reduced in these groups. However, in group HL, the levels of this protein were higher than in group H, increasing slightly in group HL over the weeks. This could be related to the consumption of tomato juice, since several studies have described changes in plasmatic cholesterol (total and its fractions) related to the consumption of tomato juice and the accumulation of lycopene in the body [17,40,41], mainly due to the inhibitory effect of lycopene on 3-hydroxy-3-methylglutaryl-CoA reductase [42].

The association of the serum concentrations of extracellular matrix components, especially serum type IV collagen 7S, measured with routine laboratory parameters, and the degree of fibrosis in NAFLD has been studied extensively [43,44,45]. It is believed that the ballooning of hepatocytes and releases of type IV collagen are the main causes of the increased serum levels, although the mechanism of this interaction is still unknown [39]. Accordingly, in our study other types of collagen have been detected in the urine samples, but without a clear behavior.

Uromodulin has been validated as a biomarker of hypertension and renal injury and has been found at higher levels in patients with hypertension, relative to healthy individuals [46]. This peptide was not detected in healthy rats, but was present in the H groups; in particular, a higher relative abundance of uromodulin was observed in group HA, since a high-fat diet is associated with disturbances of arterial pressure [47]. The consumption of tomato juice appeared to exert a positive effect on health, because—although the frequency of uromodulin in the HL group was 100%—the relative abundance was reduced significantly, by more than a half, in comparison with the HA group samples. This finding could be explained by the ability of lycopene to reduce the systolic blood pressure, as suggested by Ried and Falkler [40], or by the general improvement of endothelial function produced by lycopene [48].

Furthermore, the abundance of transketolase was considerably reduced in the H groups, especially in group HA (fold change −5.94). This enzyme participates in numerous metabolic pathways at the cellular level, such as the pentose phosphate pathway, and its activity has been reported to change in several pathologies such as diabetes. In fact, this enzyme plays an important role in the prevention of vascular damage in hyperglycemia, caused mainly by injury to the mitochondrial function due to the presence of reactive oxygen species [49]. Moreover, Boren et al. [50] showed that this enzyme was also active in the peroxisomes of liver parenchymal cells; so, hepatic damage could alter such activity. The proline-rich proteins (PRPs) are a heterogeneous group of proteins with important biological functions, such as the expression of immunomodulatory and antioxidant properties, in secondary modifications of collagen molecules and in the modulation of interactions between proteins, so they have a crucial role in cellular signal transduction pathways [51]. In rats with steatosis the relative abundance of transketolase was significantly reduced in comparison with the N and HL groups, whereas the relative abundance of PRPs was significantly increased in HA, more than in HL. The transketolase levels were especially low in rats with steatosis, probably due to the damage to mitochondria and peroxisomes caused by lipid peroxidation products. The higher levels of PRPs might be related also to NAFLD, their increasing concentrations being metabolic mechanisms to combat the oxidative stress and inflammation associated with steatosis. In fact, the rats of group HA showed higher levels of oxidative stress than rats of group HL, which recovered their redox balance due to the protection of lycopene and showed lower urinary isoprostanes levels, a lower NAD/NADH ratio and increased amounts of the intermediates in the metabolism of methionine [21]. No significant differences were observed in the biomarkers of inflammation (TNFα and IL-6) among the healthy and ill rats [24].

A similar tendency was observed for other proteins related to CVD. Extracellular superoxide dismutase (Cu-Zn) catalyzes the conversion of oxidative molecules, such as nitric oxide and the superoxide anion, thereby preventing the endothelial damage and mitochondrial dysfunction which occur in the pathogenesis of CVD [52]. Other proteins, such as collagen α-1(I) (peptide 11153), collagen α-1(II) (peptide 4970) and the fibrinogen α chain precursor (peptide 7243), have been validated as biomarkers of CVD and diabetes [53,54]. In particular, these peptides were more abundant in group HA than in groups N and HL, which could be associated with a higher risk of CVD in animals with steatosis [8,9]. These results show again the beneficial effects of the consumption of tomato juice on the amelioration of steatosis, leading to a reduction of the CVD risk.

Concerning the l-lactate dehydrogenase B chain, this enzyme is present in the mitochondria, so damage to these organelles could decrease its excretion in urine. This could explain the low levels observed in group HA [55] and, likewise, it suggests a protective effect of tomato juice consumption—according to the intermediate situation of the values of group HL in comparison with group HA and the healthy group. Mitochondria are damaged by lipid peroxidation products, so the accumulation of lycopene in the liver could protect them, improving their functionality.

It is interesting to observe that the amount of classificatory peptides detected in Week 4 was very low, compared with the other weeks. This suggests that the main changes occurred at the beginning of the study, when most of the physiological disturbances caused by the consumption of a hypercholesterolemic and high-fat diet were triggered, and also that the supplementation of lycopene improved the metabolism. However, in the fourth week, the effect of the continuous delivery of fat in the diet could not be counteracted by the supplementation of tomato juice and the accumulation of lycopene, meaning that there were no significant changes in the urinary peptide profile.

Summing up, this research provides new information about the urinary peptides that could be associated with steatosis, describing a relationship between the main proteins and the clinical evolution of this illness. In addition, and taking into consideration the amelioration of the steatosis associated with the intake of tomato juice and the accumulation of lycopene in the liver, this effect is also reflected in the changes observed in the urinary peptide profile. For this reason, the discovery of biomarkers in urine samples, which are easy to collect and have great stability, should be explored further, for early diagnosis of NAFDL as well as to determine the pathological state. Therefore, further investigations in humans are necessary to be able to create a specific urinary biomarkers panel for this disease of rising importance. In this respect, CE-MS technology enables the reproducible analysis of low molecular weight proteome, whose data can be used for diagnosis, prognosis and assessment of therapy, due to its ability for the definition and validation of biomarker patterns for a clinical application [56,57,58,59]. On the other hand, the use of other techniques, like Western blot or Enzyme-Linked ImmunoSorbent Assay (ELISA), would be interesting for the confirmation of specific proteins as clinical biomarkers. In fact, a combination of both technologies, CE-MS and immunological tests, may be the best advance toward solving yet unmet clinical needs [57,60].

## 4. Materials and Methods

### 4.1. Tomato Juice

Commercial tomato juice was provided by a local juice producer. It was obtained from an industrial standard process and commercialized in glass bottles. The juice was analyzed to determine the total content of bioactive compounds, following the methods described previously [14,15]. The contents of these compounds were: total lycopene 108 mg/kg, total phenols 284 mg/kg, free flavonoids 36 mg/kg and total folates 340 µg/kg. In addition, this tomato juice contained 14.4 mg/L of vitamin C and had a calorific value of 260 kcal/L.

### 4.2. Animals and Experimental Design

Twenty-four male Sprague-Dawley rats (8 weeks old), weighing approximately 250 g, were obtained from the Animal Facility of the University of Murcia (Murcia, Spain). The sample size was calculated using the method based on the law of diminishing return, following the procedure described by Charan and Kantharia [61]; this gave a sample size that was more than adequate. The rats were randomly divided into two groups (*n* = 12) fed ad libitum with a standard diet (N) (Teklad global 14% protein rodent maintenance diet, Harlan Laboratories, Indianapolis, IN, USA) or a hypercholesterolemic and high-fat diet (H) (Atherogenic rodent diet TD-02028, Harlan Laboratories, Indianapolis, IN, USA) and water during a 2-week adaptation period. Afterwards, each group was randomly sub-divided into two other groups (*n* = 6) and these were placed individually in metabolic cages, yielding the following groups: standard diet and water (NA), standard diet and tomato juice (NL), hypercholesterolemic and high-fat diet and water (HA) and hypercholesterolemic and high-fat diet and tomato juice (HL). The rats were maintained under controlled conditions of temperature (22 °C) and air humidity (55%), with a 12-h light-dark cycle, during all the study. Urine samples were collected weekly for four weeks and collection was performed over a period of 24 h, giving a total of 96 samples. At the end of the study the rats were euthanized and blood and liver samples were collected. All samples were stored at −80 °C until the analytical procedures were carried out. The study was carried out at the experimental Animal Facility of the University of Murcia (Murcia, Spain; Registration number: REGA ES 300305440012), in strict accordance with the recommendations of the European Union regarding animal experimentation (Directive of the European Council 2010/63/UE). The protocol was approved by the Ethics Committee of the University of Murcia and the local government (Murcia Autonomous Government, Department of Agriculture, Fisheries and Livestock, permit number: A1320140701, permitted on 23 July 2014).

### 4.3. NAFLD Confirmation

In the rats fed with diet H (HA and HL groups), NAFLD was confirmed by analyzing the biochemical parameters (total cholesterol, HDL-cholesterol, LDL-cholesterol, total triglycerides) and hepatic enzymes (ALT and AST) and by histological examination of the liver using hematoxylin and eosin stain. All analyses were carried out in the Veterinary Hospital of the University of Murcia.

### 4.4. Sample Preparation

Rat urine was thawed immediately before use and a 0.7 mL aliquot of urine was diluted with 0.7 mL of 2 M urea and 10 mM NH_4_OH containing 0.02% sodium dodecyl sulfate (all from Sigma-Aldrich, Dorset, UK), as described by Albalat et al. [28]. Purified peptides were lyophilized and stored at 4 °C until analysis. 

### 4.5. Protein Estimation

The protein concentration was quantified in urine samples using the bicinchoninic acid (BCA) assay Uptima, from Interchim (Montluçon, France). Freeze-dried aliquots were re-suspended in HPLC-grade water to reach a concentration of 2 µg/µL, shortly before CE-MS analyses as described by Mullen et al. [36].

### 4.6. Capillary Electrophoresis Coupled to Mass Spectrometry (CE-MS) Analysis

The CE-MS analyses were performed using a P/ACE™ MDQ capillary electrophoresis system (Beckman Coulter, Fullerton, CA, USA), with a 90-cm, 50-µm I.D., fused-silica, non-coated capillary (New Objective, Woburn, MA, USA), coupled online to a micrOTOF (time-of-flight) mass spectrometer (Bruker Daltonic, Bremen, Germany). The electro-ionization sprayer (Agilent Technologies, Santa Clara, CA, USA) was grounded and the ion spray interface potential was set between −4 and −4.5 kV, as described previously [28]. Sheath-flow liquid, including 2-propanol (30% *v*/*v*) and formic acid (0.4% *v*/*v*) (both from Sigma-Aldrich, Dorset, UK) diluted with HPLC-grade water, was applied coaxially at a running speed of 20 µL/h. A solution of 20% acetonitrile in HPLC-grade water, supplemented with 0.94% formic acid, was used as running buffer.

The MS spectra were recorded over an *m/z* range of 350–3000 and accumulated every 3 s. The accuracy, precision, selectivity, sensitivity, reproducibility and stability of the CE-MS measurements are described in Theodorescu et al. [56].

### 4.7. CE-Data Processing

The MS ion peaks were processed using MosaiquesVisu software (Mosaiques Diagnostics, Hannover, Germany), which includes peak picking, deconvolution and deisotoping [62]. The CE migration time and peak intensity were subsequently normalized using internal polypeptide standards [56], to allow compilation and comparison of samples. The resulting peak list characterizes each polypeptide by its molecular mass (0.8–30 kDa), normalized CE migration time (min) and normalized signal intensity (ion counts). The normalized signal intensity was used as a measure of relative abundance. All the polypeptides detected were deposited, matched and annotated in a Microsoft SQL database [36], to allow the identification of proteins from the peptide sequences obtained from the urine samples. Polypeptides from different samples were considered identical if the mass deviation was lower than ±50 ppm and the migration time lower than 2 min.

### 4.8. Statistical Analyses for Biochemical Parameters

One-way ANOVA and a post hoc Tukey test were carried out to determine the differences among the four experimental groups regarding the different biochemical parameters. The data are expressed as the mean ± SE and the significance level was *p* < 0.05. The statistical analyses were performed with the IBM Statistical Package for the Social Sciences (SPSS), version 19.0 (IBM, New York, NY, USA).

### 4.9. Statistical Analysis for Biomarker Definition

A sparse partial least squares-discriminant analysis (sPLS-DA) was applied to explore the associations between peptides and treatments, using the mixOmics package [63,64] of R [65]. The sPLS-DA method combines the ability of PLS to extract latent variables from matrices with a very high number of variables and a low number of cases (typical of -omics) with the ability of DA to separate groups of different treatments.

Exploratory analyses showed that the peptide profiles of the rats of diet N were very similar, independently of the administration of tomato juice; therefore, we built three different types of model: (i) models comparing four groups (NA, NL, HA, HL) within a week; (ii) models comparing three groups (N, HA, HL) within a week; and (iii) a model comparing all groups and weeks simultaneously (in practice, a model of 16 groups, one per treatment and week).

The original database includes 888 peptides. From the point of view of biomarker identification, an analysis including all of these peptides is not useful; therefore, the objective of the sPLS-DA was to find a model that maximizes the correct classification (minimizes the error rate of the classification) of treatments based on their peptide profile while minimizing the number of peptides used. A basic model was defined by its number of components (latent variables) and the number of peptides in the model. The number of components ranged from 1 to *n*-1 groups to be classified, as *n* groups may be segregated by *n*-1 latent variables. The maximum number of peptides to be included in a model was arbitrarily set to 50, considering that a biomarker set >50 peptides is unnecessarily complicated for routine clinical application. Then, a total of 1750 models were tested, resulting from all the possible combinations of grouping, number of components and number of peptides (Table A1).

From this pool of 1750 models the best models for the identification of biomarkers were selected on the basis of the lowest classification error rate, estimated by cross-validation. Briefly, for each basic type of model the rats in the sample were divided into 10 sub-groups: nine of these were used to estimate the model and the rats in the excluded sub-group were classified into the treatments according to this model. Misclassification was the error rate. This was repeated 10 times per basic model (excluding one sub-group per step) in order to calculate a mean error rate per model.

Models based on three groups within a week showed the lowest mean error rates (0.17 to 0.25, depending on the week), performing much better than models based on four groups within a week or the model of 16 groups with all treatments and weeks analyzed simultaneously. Then, we selected the five “best” models (lowest error rates) in each week to screen them for biomarkers. Peptides with a minimum load of ±0.15 in all five models were named as potential biomarkers. Load represents the correlation between the peptide and the component (latent variable), which optimizes the separation of treatments. Furthermore, in order to minimize the biomarker set and maximize its usefulness, we decided, as a threshold, that a peptide selected as a biomarker should be detected also in >30% of the samples in at least one group.

## Figures and Tables

**Table 1:** Plasma biochemical parameters and hepatic enzymes activity analyzed in the four experimental groups at the end of the intervention period ^1^.

Parameters	NA	NL	HA	HL
Total Cholesterol (mg/dL)	99 ± 4.1 ^b^	81 ± 6.5 ^b^	167 ± 14 ^a^	162 ± 14 ^a^
LDL-cholesterol (mg/dL)	26 ± 1.4 ^b^	17 ± 2.4 ^b^	94 ± 11 ^a^	96 ± 8.2 ^a^
HDL-cholesterol (mg/dL)	54 ± 2.3 ^a^	44 ± 3.5 ^a,b^	40 ± 2.1 ^b^	49 ± 2.7 ^a,b^
Triglycerides (mg/dL)	80 ± 4.6 ^b^	78 ± 8.3 ^b^	121 ± 7.1 ^a^	98 ± 14 ^a,b^
ALT (U/L)	32 ± 1.9 ^b^	41 ± 2.9 ^b^	79 ± 11 ^a^	79 ± 12 ^a^
AST (U/L)	68 ± 7.1 ^b^	67 ± 4.9 ^b^	150 ± 7.6 ^a^	143 ± 20 ^a^

^1^ Values are expressed as mean ± SE. Different letters ^(a,b)^ show significant statistical differences between groups after carrying out one-way ANOVA (*p* < 0.05). NA: standard diet and water; NL: standard diet and tomato juice; HA: hypercholesterolemic and high-fat diet and water; HL: hypercholesterolemic and high-fat diet and tomato juice; ALT: Alanine transaminase; AST: Aspartate transaminase.

**Table 2:** Peptides with greater discriminant power between groups, which changed over the weeks. na: not applicable.

Week	Peptide ID	Peptide Mass (Da)	CE Time (min)	Sequence Peptide	Protein Identity	Mean Rel ab N	Freq N	Mean Rel ab HA	Freq HA	Mean Rel ab HL	Freq HL	Fold Change HA	Fold Change HL
1	7035	1197.6	37.2	DSYVGDEAQSK	Actin. α skeletal muscle	482.2	91.7	195.8	16.7	0.0	0.0	−2.46	na
1	10615	1433.7	39.2	GEVGPpGPpGPAGEKG	Collagen α-1(I) chain precursor	506.7	100.0	164.9	50.0	483.4	40.0	−3.07	−1.05
1	13476	1652.8	40.9	GEpGEpGQTGPAGSRGPA	Collagen α-2(I) chain	452.8	91.7	0.0	0.0	67.6	20.0	na	−6.70
1	13640	1668.9	35.1	WGKVNPDDVGGEALGR	Hemoglobin subunit beta-1	0.0	0.0	5343.6	100.0	3360.0	100.0	na	na
1	17766	2088.1	36.3	AGRpGEVGPpGPpGPAGEKGSpG	Collagen α-1(I) chain precursor	102.5	100.0	0.0	0.0	93.6	20.0	na	−1.09
1	7744	1240.6	31.4	AGYQRELNYK	α-1-inhibitor 3 precursor	0.0	0.0	4339.0	100.0	2920.1	100.0	na	na
1	14576	1759.9	34.0	SADTLWDIQKDLKDL	l-lactate dehydrogenase B chain	615.4	91.7	67.1	66.7	66.0	60.0	−9.17	−9.32
1	13862	1691.9	35.2	DGKTGPpGPAGQDGRPGp	Collagen α-1(I) chain	336.8	100.0	89.1	16.7	250.8	60.0	−3.78	−1.34
1	11124	1470.8	32.6	FSHIDVSPGSAQVK	Hemoglobin subunit α-1/2	39.0	8.3	1755.1	100.0	1770.7	100.0	45.04	45.44
1	10647	1435.7	31.1	SLKGFSQQTQQKG	Seminal vesicle secretory protein 2 precursor	838.1	75.0	2676.8	100.0	2623.0	80.0	3.19	3.13
1	13451	1649.8	41.3	TDVTQQLNTLFQDK	Apolipoprotein A-IV	1863.1	100.0	847.2	100.0	1080.5	100.0	−2.20	−1.72
1	11115	1469.7	39.3	GSpGAPGApGHpGPpGP	Collagen α-1(III) chain	271.6	91.7	25.4	16.7	0.0	0.0	−10.70	na
2	10013	1388.7	39.2	RpGEVGPpGPpGPAG	Collagen α-1(I) chain	3710.4	100.0	892.4	80.0	1133.4	100.0	−4.16	−3.27
2	13862	1691.9	35.2	DGKTGPpGPAGQDGRPGp	Collagen α-1(I) chain	466.0	100.0	0.0	0.0	306.4	16.7	na	−1.52
2	14941	1795.9	41.6	GETGNKGEpGSAGAQGPpGP	Collagen α-2(I) chain	146.7	100.0	0.0	0.0	14.9	16.7	na	−9.88
2	8057	1260.7	38.4	RpGEVGPpGPpGP	Collagen α-1(I) chain	675.8	100.0	0.0	0.0	271.4	16.7	na	−2.49
2	24835	3124.6	32.9	DELVRDKPYGPKVSGGSFGEEASEEISSR	Seminal vesicle protein 2 precursor	0.0	0.0	1421.0	100.0	947.8	100.0	na	na
2	17689	2081.0	26.9	DGPpGRDGQpGHKGERGYpG	Collagen α-2(I) chain	1593.7	100.0	615.3	80.0	829.7	100.0	−2.59	−1.92
2	11667	1511.7	39.3	AGPpGPpGpPGSIGHpG	Procollagen. type IX. α 3 (predicted). isoform CRA a	125.4	91.7	0.0	0.0	0.0	0.0	na	na
2	9863	1378.7	39.2	ApGEDGRpGPpGPQ	Collagen α-1(II) chain	825.0	100.0	705.3	20.0	281.0	83.3	−1.17	−2.94
2	10377	1414.7	38.7	LYQAEAFIADFK	Contrapsin-like protease inhibitor 3 precursor	324.1	100.0	75.4	40.0	0.0	0.0	−4.30	na
2	13429	1647.8	40.5	GApGPAGPAGERGEQGPAG	Collagen α-1(I) chain	101.1	16.7	0.0	0.0	762.1	66.7	na	7.54
2	13543	1659.8	39.7	GAPGAKGNVGppGEPGPpG	α 4 type V collagen	156.0	100.0	307.6	20.0	260.1	100.0	1.97	1.67
2	15097	1811.9	42.1	GGAGPpGPEGGKGPAGPpGPpG	Collagen α-1(III) chain	188.6	66.7	535.0	100.0	0.0	0.0	2.84	na
2	7243	1210.6	38.0	QLQEGPPEWK	Fibrinogen α chain precursor	99.2	66.7	499.0	80.0	101.7	16.7	5.03	1.03
2	8984	1320.7	38.2	GPpGENGKPGEpGP	Collagen α-1(III) chain	861.3	100.0	798.5	80.0	1560.3	100.0	−1.08	1.81
2	2712	933.5	47.2	PGpAGPpGPVG	Collagen α-1(II) chain	13.3	33.3	0.0	0.0	27.4	66.7	na	2.06
2	4970	1066.5	36.7	GEDGRpGPpGP	Collagen α-1(II) chain	408.7	75.0	984.6	100.0	377.9	33.3	2.41	−1.08
2	9955	1384.7	32.7	VDLADRLDLVEK	Extracellular superoxide dismutase (Cu-Zn) precursor	415.5	66.7	2275.0	100.0	266.7	33.3	5.47	−1.56
2	10240	1405.7	39.1	GLpGPAGPpGEAGKpG	Collagen α-1(I) chain	1005.1	91.7	1430.2	60.0	1830.2	100.0	1.42	1.82
2	11153	1472.7	49.2	GPpGPSGNAGppGPpGP	Collagen α-1(I) chain	0.0	0.0	1478.7	80.0	170.1	33.3	na	na
2	10124	1396.7	31.8	SRLAPLAEGVQEK	Apolipoprotein A-IV	132.4	66.7	378.2	80.0	254.3	16.7	2.86	1.92
2	17855	2096.1	35.8	FQDKLGNINTYADDLQNK	Apolipoprotein A-IV precursor	0.0	0.0	1731.4	100.0	642.7	100.0	na	na
2	9540	1354.6	48.8	EpGTTGPpGTAGPQG	Collagen α-2(I) chain	1212.4	100.0	382.3	40.0	697.7	50.0	−3.17	−1.74
2	12640	1584.8	34.0	DGQPGAKGEpGDTGVKG	Collagen α-1(I) chain	323.8	100.0	101.6	100.0	120.8	66.7	−3.19	−2.68
2	13476	1652.8	40.9	GEpGEpGQTGPAGSRGPA	Collagen α-2(I) chain	541.2	91.7	304.1	20.0	0.0	0.0	−1.78	na
2	14976	1799.9	41.5	PPTGPFVEPPDLFFLK	Proline-rich protein	441.3	83.3	51010.5	100.0	14119.9	100.0	115.59	32.00
2	4768	1051.5	36.5	TGVKGDAGPPGP	Collagen α-1(I) chain	134.6	100.0	106.8	60.0	220.0	100.0	−1.26	1.63
2	20632	2428.3	26.6	DGILGRDTLPHEDQGKGRQLHS	Contrapsin-like protease inhibitor 1 precursor	84.2	16.7	254.8	80.0	983.1	100.0	3.03	11.68
2	15844	1887.9	40.9	AKALYQAEAFTADFQQS	Contrapsin-like protease inhibitor 6 precursor	62.1	33.3	450.6	80.0	135.7	16.7	7.25	2.18
2	4524	1035.5	35.6	GPpGPEGGKGPA	Collagen α-1(III) chain	461.9	58.3	471.4	60.0	896.5	100.0	1.02	1.94
3	13476	1652.8	40.9	GEpGEpGQTGPAGSRGPA	Collagen α-2(I) chain	624.1	100.0	171.8	33.3	192.1	50.0	−3.63	−3.25
3	14544	1756.9	41.9	GNFIDQTRVLNLGPIT	Uromodulin	0.0	0.0	2190.6	100.0	976.5	100.0	na	na
3	10648	1435.7	39.6	SpGSPGPDGKTGPpGP	Collagen α-1(I) chain precursor	1141.3	100.0	147.2	16.7	0.0	0.0	−7.75	na
3	8116	1263.6	32.3	ISSDLDGHPVPK	Transketolase	392.6	100.0	66.1	50.0	104.0	100.0	−5.94	−3.77
3	13451	1649.8	41.3	TDVTQQLNTLFQDK	Apolipoprotein A-IV	1740.8	100.0	509.9	100.0	922.7	100.0	−3.41	−1.89
3	17002	2008.0	31.2	DGESGRpGRpGERGLpGPpG	Collagen α-1(III) chain	507.3	100.0	68.9	33.3	136.2	100.0	−7.36	−3.72
3	14576	1759.9	34.0	SADTLWDIQKDLKDL	l-lactate dehydrogenase B chain	683.8	100.0	31.0	16.7	150.3	83.3	−22.03	−4.55
3	16532	1960.0	35.5	RpGEVGPpGPpGPAGEKGSpG	Collagen α-1(I) chain	170.7	100.0	0.0	0.0	97.1	50.0	na	−1.76
3	17766	2088.1	36.3	AGRpGEVGPpGPpGPAGEKGSpG	Collagen α-1(I) chain precursor	164.6	100.0	20.0	16.7	79.9	50.0	−8.22	−2.06
3	13970	1702.0	22.8	ELEKETKKETKKDP	α-1-acid glycoprotein precursor	0.0	0.0	75.3	50.0	303.6	100.0	na	na
3	19332	2260.0	26.3	MADEAASEAHQEGDTRTTKRG	Fibrinogen α chain precursor	28.4	9.1	10.6	16.7	47.4	83.3	−2.69	1.67
3	4486	1032.5	36.9	pGPpGPRGPpG	Collagen α-1(XVII) chain	12.4	36.4	8.1	16.7	21.3	83.3	−1.52	1.72
3	8197	1269.6	39.2	GFpGAAGRTGPpGP	Collagen α-2(I) chain	30.4	45.5	130.6	83.3	13.9	50.0	4.30	−2.19
3	5870	1124.6	30.8	DLEDVAGHGGR	Cd99 protein	10.5	9.1	0.0	0.0	21.0	50.0	na	1.99
3	21145	2497.4	38.7	DLPGQQPVSEQAQQKLPPLALLK	Serine (Or cysteine) peptidase inhibitor. clade F. member 2	34.6	9.1	71.2	33.3	161.9	83.3	2.06	4.69
3	18962	2216.1	26.6	DHGKELSSSLGPALVDKAPAPK	Similar to CG7896-PA	0.0	0.0	22.4	33.3	36.1	83.3	na	na
3	17050	2013.0	36.7	DEQYPDATDEDLTSRMK	Osteopontin precursor	26.9	54.5	58.8	33.3	57.7	100.0	2.19	2.15
3	5198	1079.5	47.6	GQpGPpGPpGTA	Collagen α-1(III) chain	9.2	63.6	0.0	0.0	15.0	66.7	na	1.62
4	6975	1194.6	37.7	SpGPDGKTGPpGP	Collagen α-1(I) chain	12172.0	100.0	6619.6	100.0	6543.0	100.0	−1.84	−1.86
4	11644	1509.8	27.3	GLpGpKGDRGDAGPKG	Collagen α-1(I) chain precursor	52.9	83.3	50.7	16.7	187.6	100.0	−1.04	3.55

Rel ab: relative abundance; CE: capillary electrophoresis.

## Appendix A

**Table A1:** Representation of all the possible combinations of grouping, number of components and number of peptides, resulting in 1750 models. na: not applicable.

Grouping	Weeks	Number of Components	Number of Peptides	Number of Combinations	Total
16 groups (NAw1, NLw1, HAw1, HLw1, NAw2,…, HLw4)	na	1–15	1–50	1 × 15 × 50	750
4 groups (NA, NL, HA, HL)	4	1–3	1–50	4 × 3 × 50	600
3 groups (N, HA HL)	4	1–2	1–50	4 × 2 × 50	400
					1750
